# Increased expression of kindlin‐2 is correlated with hematogenous metastasis and poor prognosis in patients with clear cell renal cell carcinoma

**DOI:** 10.1002/2211-5463.12063

**Published:** 2016-06-01

**Authors:** Meisi Yan, Lei Zhang, Yiqi Wu, Lei Gao, Weiwei Yang, Jing Li, Yubing Chen, Xiaoming Jin

**Affiliations:** ^1^ Department of PathologyHarbin Medical UniversityChina; ^2^ Electron Microscopy CentreHarbin Medical UniversityChina; ^3^ Department of RadiotherapySecond Hospital of Jilin UniversityChangchunChina

**Keywords:** clear cell renal cell carcinoma, hematogenous metastasis, kindlin‐2, prognosis

## Abstract

Kindlin‐2 is involved in activating the integrin signaling pathway which plays an important role in regulating cancer cell invasion. However, the role of kindlin‐2 may vary among cancer types. The aim of this study was to explore the possible association between kindlin‐2 and clear cell renal cell carcinoma (ccRCC), and its potential role in the prognosis of ccRCC. Immunohistochemistry assays were used to examine kindlin‐2 expression levels in cancer tissues obtained from 336 patients with ccRCC. The correlation between kindlin‐2 expression levels and pathologic variables was then analyzed. In addition, the association between kindlin‐2 expression levels and survival time was analyzed by Kaplan–Meier survival curves and log‐rank tests. Of 336 ccRCC patients, 199 had high levels of kindlin‐2 expression, while 137 had low kindlin‐2 expression levels. Patients at a late stage of ccRCC (stage III or IV) were more likely to have high kindlin‐2 expression levels than those at an early stage (stage I or II) (χ^2^ = 4.72, *P* = 0.03). Patients with high levels of kindlin‐2 expression had higher risk of hematogenous metastasis (χ^2^ = 6.70, *P* = 0.01) than those with low levels of kindlin‐2 expression. In addition, the survival time was significantly shorter for patients with high levels of kindlin‐2 expression than for those with low levels of kindlin‐2 expression (*P* = 0.001 for overall survival [OS] and *P* = 0.002 for disease‐free survival [DFS]). Multivariate survival analysis based on the Cox proportional hazards model showed that high kindlin‐2 expression levels had a hazard risk (HR) of 1.76 for OS (95% CI 1.19–2.62, *P* = 0.005) and an HR of 1.47 for DFS (95% CI = 1.05–2.06, *P* = 0.026). By comparison, lymph node metastasis had an HR of 1.48 for OS (95% CI 1.04–2.10, *P* = 0.029) and an HR of 1.41 for DFS (95% CI 1.04–1.93, *P* = 0.029). This study provided strong evidence that increased kindlin‐2 expression might be involved in promoting tumor invasiveness and leading to a poor prognosis of ccRCC.

**Abbreviations**
ccRCC: Clear cell renal cell carcinoma
cell–ECM: cell–extracellular matrix
DFS: disease‐free survival
HR: hazard risk
IHC: immunohistochemistry
OS: overall survival
RCC: renal cell carcinoma

Renal cell carcinoma (RCC) is the 10th most common cause of cancer‐related deaths worldwide, and 30% of all cases present metastases at the time of initial diagnosis owing to the generally mild clinical signs at early stages 1. Metastasis represents a great challenge for patients with clear cell RCC (ccRCC), as metastasis is always associated with poor prognosis. Despite the rapid development of new treatments, the 5‐year survival rate for metastatic ccRCC remains under 10% 2. Therefore, there is an urgent need for identification of factors involved in metastasis and for the development of early metastasis detection techniques, which is the best approach to reduce ccRCC‐induced morbidity and to improve treatment.

One of the hallmarks of metastatic cancer cells is increased invasiveness. It has been reported that integrins and cell‐extracellular matrix (cell‐ECM) adhesion proteins play important roles in the regulation of cancer cell invasion 3, 4, 5. Kindlins (including kindlin‐1, kindlin‐2, and kindlin‐3) are adaptor proteins that bind to the cytoplasmic tails of integrins 6. Kindlin‐2 is involved in activating integrins and regulating cell–matrix adhesion 7, 8 and acts as a component of cell‐ECM adhesion complexes by directly interacting with the adhesion complex protein integrin‐linked kinase (ILK), migfilin, and integrins 9, 10, 11. Owing to the potential role of kindlin‐2 in cancer cell invasion through regulation of the integrin signaling pathway, several studies have evaluated the association between kindlin‐2 expression and different types of cancers, including breast cancer, mesenchymal cancer, malignant mesothelioma, gastric cancer, bladder cancer, and leiomyoma 12, 13, 14, 15, 16, 17. However, controversial roles of kindlin‐2 have also been reported in different types of cancer cells. Kindlin‐2 may play a positive role in promoting the invasion of breast cancer, malignant mesothelioma, gastric cancer, and bladder cancer 13, 15, 16, 17. In addition, a possible role of kindlin‐2 in regulating mesenchymal cancer cell invasion was proposed based on the fact that kindlin‐2 expression was significantly lower in uterine leiomyoma cells that adopted a mesenchymal morphology than in less invasive uterine leiomyoma cells 12. On the contrary, a suppressive role of kindlin‐2 has also been observed in invasive mesenchymal cancer cells 14. Therefore, the role of kindlin‐2 may vary among cancer types, and the potential association of kindlin‐2 expression with ccRCC invasiveness and prognosis has yet to be elucidated. This study was designed to explore the possible relationship between kindlin‐2 and ccRCC, and its potential value in prognosis of ccRCC.

## Materials and methods

### Patients and specimens

A total of 336 patients with ccRCC were included in this study. Their demographic and clinicopathological characteristics are summarized in Table 1. Of these 336 patients, aged between 32 and 75, 240 were male and 96 were female. Lymph node metastasis was present in 134 patients and hematogenous metastasis was present in 67 patients. The follow‐up time ranged from 10 to 60 months. Primary cancers were evaluated in accordance with the seventh edition of the American Joint Committee on Cancer staging system (TNM). The pathological grading of the tissue samples was performed by two senior pathologists using the Fuhrman grading system. The ccRCC tissue samples used in this study were retrieved from the tissue biobanks at the Second Affiliated Hospital and the Third Affiliated Hospital, Harbin Medical University, Harbin, China. This study was approved by the Ethics Committee of Harbin Medical University and conformed to the requirements of the Declaration of Helsinki.

**Table 1:** Clinical characteristics of 336 patients with ccRCC

Characteristics	Number of cases (%)
Gender	
Male	240 (71.4)
Female	96 (28.6)
Age (years)	
< 65	177 (52.7)
≥ 65	159 (47.3)
Tumor size (cm)	
< 4	176 (52.4)
≥ 4	160 (47.6)
Grade	
I–II	107 (31.8)
III–IV	229 (68.2)
cTNM	
I–II	124 (36.9)
III–IV	212 (63.1)

T1–T2	167 (49.7)
T3–T4	169 (50.3)
Lymph nodes metastasis	
Absence	202 (60.1)
Presence	134 (39.9)
Hematogenous metastasis	
Absence	269 (80.1)
Presence	67 (19.9)

### Tissue microarray and immunohistochemistry analysis

Tissue cores with a 3‐μm thickness were selected from individual paraffin‐embedded tissue blocks, punched out using a punch machine and placed into a recipient block. All ccRCC blocks were cut to 4‐μm thick sections using a microtome and arrayed in triplicate. Each tissue core was assigned a unique tissue microarray location number, which was linked to a database containing other clinical and pathological information.

Kindlin‐2 expression in tissue sections was measured by immunohistochemistry (IHC) for all 336 ccRCC patients. The paraffin‐embedded tissues were cut into 4‐mm sections and dried at 70–73 °C for 3 h. After deparaffinization and rehydration, the tissue sections were washed three times in phosphate‐buffered saline (PBS, 3 × 3 min), and were then treated with 3% H_2_O_2_ in the dark for 5–20 min. After being washed in distilled water, the tissue sections were washed in PBS (3 × 5 min). Antigen retrieval was performed in citrate buffer (pH 6.0). Each section was treated with 50–100 μL of primary antibody, and then incubated overnight at 4 °C with a solution of mouse‐anti‐human kindlin‐2 monoclonal IgG antibodies (ab117962; Abcam, Cambridge, MA, USA) diluted at 1:100. After being washed in PBS (3 × 5 min), each section was incubated for 30 min at room temperature with 50–100 μL of secondary antibody. After being washed in PBS (3 × 5 min), each section was treated for 3–10 min at room temperature with 50–100 μL of diaminobenzidine working solution and then washed in distilled water. Double‐blind analysis was performed on all samples by two senior pathologists.

### Scoring system

The relative levels of kindlin‐2 expression were evaluated by semi‐quantitation. Briefly, the intensity of positive anti‐kindlin‐2 IgG staining in 10 randomly selected high‐power fields (magnification ×400) was evaluated independently by two investigators. The percentage of positively stained tumor cells in a given field was scored as 0 (0% of cells positively stained), 1 (up to 10% of cells positively stained), 2 (10–50% of cells positively stained), or 3 (over 50% of cells positively stained). The staining intensity in a given field was scored as 0 (no staining), 1 (weak staining, appearing as light yellow), 2 (moderate staining, appearing as yellowish‐brown), or 3 (strong staining, appearing as brown). The staining index (SI) of individual sections was calculated as the averaged staining intensity score multiplied by the score of the proportion of stained cells. The cut‐off value for anti‐kindlin‐2 IgG staining was determined by measuring heterogeneity. Accordingly, an SI of 4 (cut‐off value) was used to distinguish between low (< 4) and high (≥ 4) levels of kindlin‐2 expression. Samples with discrepancies in the staining scores were reviewed by the two investigators and a senior pathologist until a consensus was reached. Finally, the staining assessment and classification of tumors according to the levels of kindlin‐2 expression demonstrated perfect interrater reliability.

### Statistical analysis

All categorical data were analyzed by the chi‐squared (χ^2^) test to compare the kindlin‐2 expression levels between subgroups of ccRCC patients. The Kaplan–Meier survival curve and log‐rank tests were applied to estimate overall survival (OS) times. Univariate and multivariate regression analyses based on the Cox proportional hazards model were used to identify potential factors affecting the survival time, which was expressed as the hazard risk (HR) with 95% confidence interval (CI). All statistical analyses were performed using the spss 19.0 software (SPSS Inc., Chicago, IL, USA), and *P* ≤ 0.05 was considered statistically significant.

## Results

**Figure 1:**
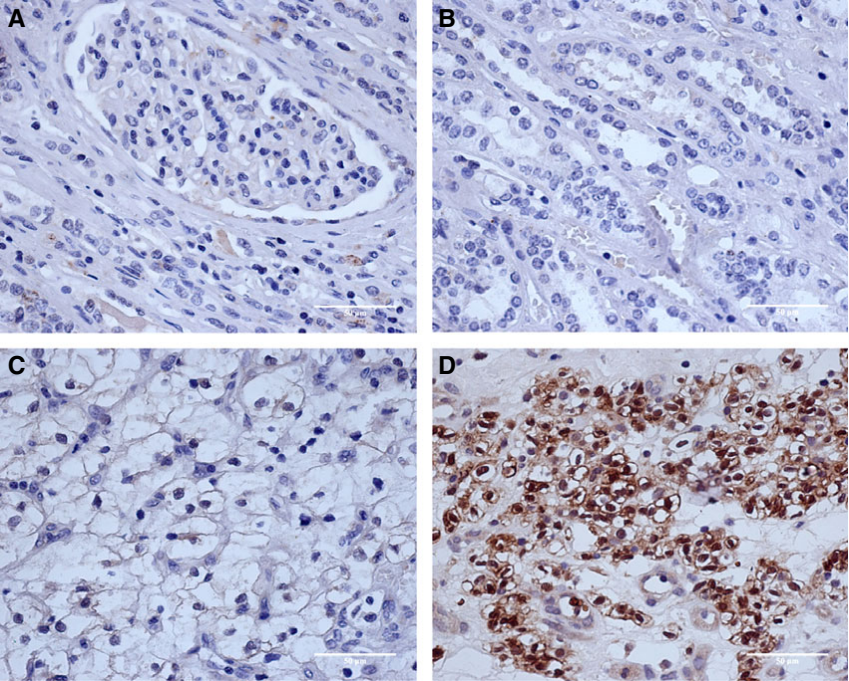
Immunohistochemistry analysis of kindlin‐2 expression. (A) Negative kindlin‐2 expression in adjacent renal cortex tissues (H&E, ×200); (B) negative kindlin‐2 expression in adjacent renal medulla tissues (H&E, ×200); (C) low kindlin‐2 expression in ccRCC cases without hematogenous metastasis (H&E, ×200); (D) high kindlin‐2 expression in ccRCC cases with hematogenous metastasis (H&E, ×200).

Kindlin‐2 expression was determined by IHC staining of 336 tissue samples from ccRCC patients. Kindlin‐2 was found to be highly expressed in ccRCC cases with hematogenous metastasis, and kindlin‐2 positive staining was mainly localized in the cell nucleus (Fig. 1). As shown in Table 2, 199 patients (59.2%) had high levels of kindlin‐2 expression while the remaining patients (40.8%) had low levels of kindlin‐2 expression. Patients at a late stage of ccRCC (stages III or IV) were more likely to have high kindlin‐2 expression levels than those at an early stage (stages I or II) (χ^2^ = 4.72, *P* = 0.03). Furthermore, patients with high kindlin‐2 expression levels had a higher risk of hematogenous metastasis (χ^2^ = 6.70, *P* = 0.01) than those with low kindlin‐2 expression levels. High levels of kindlin‐2 expression were seen in adjacent normal kidney tissues for only 35 of 336 patients (10.42%), and in cancer tissues for 199 of 336 patients (59.2%) (*P* = 0.001, Fig. 1).

**Table 2:** Association of Kindlin‐2 expression with clinical‐pathological characteristics from patients with ccRCC

Characteristics	*N*	Kindlin‐2		χ^2^	*P*
		Low (137)	High (199)		
Age (years)					
< 65	177	78	99	1.68	0.195
≥ 65	159	59	100		
Grade					
I–II	107	51	56	3.09	0.079
III–IV	229	86	143		
Tumor size (cm)					
< 4	176	80	96	3.35	0.067
≥ 4	160	57	103		
cTNM					
I–II	124	60	64	4.72	0.0301
III–IV	212	77	135		

T1–T2	167	70	97	0.18	0.672
T3–T4	169	67	102		
Lymph nodes metastasis					
Absence	202	86	116	0.68	0.41
Presence	134	51	83		
Hematogenous metastasis					
Absence	269	119	150	6.7	0.0102
Presence	67	18	49		

TNM classification for primary tumor (T), regional lymph node metastasis (N) and distant metastasis (M) was applied in accordance with the 7th edition of the American Joint Committee on Cancer staging system.

**Figure 2:**
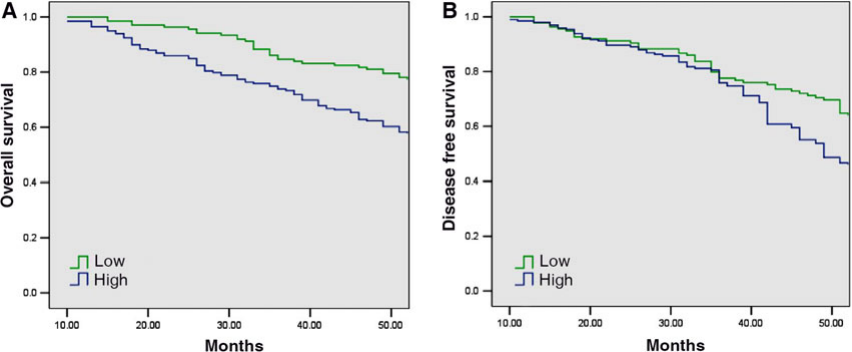
Kaplan–Meier analysis of the association between overall survival (OS) or disease‐free survival (DFS) and kindlin‐2 expression levels in ccRCC patients. (A) Kaplan–Meier analysis of the association between OS and kindlin‐2 expression levels in ccRCC patients (*P* = 0.005). (B) Kaplan–Meier analysis of the association between DFS and kindlin‐2 expression levels in ccRCC patients (*P* = 0.026). *P* value was calculated by the log‐rank test.

As shown in Fig. 2A,B, the OS time was significantly shorter in patients with high levels of kindlin‐2 expression than those with low levels of kindlin‐2 expression (47.57 vs. 53.97 months, *P* = 0.001). The disease‐free survival (DFS) time was also significantly shorter in patients with high levels of kindlin‐2 expression than those with low levels of kindlin‐2 expression (46.59 vs. 50.61 months, *P* = 0.002). Multivariate survival analysis based on the Cox proportional hazards model showed that high kindlin‐2 expression had an HR of 1.76 for the OS time (95% CI 1.19–2.62, *P* = 0.005) and an HR of 1.47 for the DFS time (95% CI 1.05–2.06, *P* = 0.026). By comparison, lymph node metastasis had an HR of 1.48 (95% CI 1.04–2.10, *P* = 0.029) for the OS time and an HR of 1.41 for the DFS time (95% CI 1.04–1.93, *P* = 0.029) (Table 3).

**Table 3:** Multivariate analysis of 5‐year overall survival (OS) and disease‐free survival (DFS) rates

Variable	OS			DFS		
	HR (95%CI)	Wald	*P*	HR (95%CI)	Wald	*P*
Kindlin‐2: high vs. low	1.76 (1.19–2.62)	7.830	0.005	1.47 (1.05–2.06)	4.982	0.026
Grade: III–IV vs. I–II	0.94 (0.65–1.37)	0.083	0.756	1.24 (0.88–1.75)	1.438	0.227
Age: ≥ 65 vs. < 65	1.04 (0.73–1.48)	0.023	0.835	1.19 (0.87–1.64)	1.155	0.285
Gender: male vs. female	1.17 (0.78–1.74)	0.528	0.453	0.88 (0.63–1.23)	0.575	0.445
Hematogenous: positive vs. negative	1.52 (1.01–2.29)	3.987	0.046	1.79 (1.25–2.56)	10.08	0.002
Node: positive vs. negative	1.48 (1.04–2.10)	4.574	0.029	1.41 (1.04–1.93)	4.766	0.029
Size: ≥ 4 vs. < 4	1.03 (0.72–1.48)	0.038	0.857	0.99 (0.72–1.35)	0.009	0.925
TNM: III–IV vs. I–II	0.94 (0.64–1.36)	0.138	0.724	1.15 (0.82–1.60)	0.625	0.429

## Discussion

Kindlin‐2 is a member of the kindlin protein family, which functions as an integrin‐binding scaffolding protein concentrated at the intracellular face of cell‐ECM adhesions 18. Kindlin‐2 has been found to play a role in embryonic development, cardiac development, and even cancer 19. To our knowledge, this is the first report on the association between kindlin‐2 expression and prognosis of ccRCC. This study found that a large proportion of ccRCC patients had high levels of kindlin‐2 expression, which was associated with advanced tumor stages and hematogenous metastasis. Significant staining of Kindlin‐2 was in the nucleus, which may be associated with its role in the TGFB1 and integrin signaling pathways. Kindlin‐2 protein stabilizes active CTNNB1 and regulates gene transcription mediated by CTNNB1 and TCF7L2/TCF4 in the Wnt signaling pathway 20. In addition, there was a significant association between kindlin‐2 expression levels and survival times (OS and DFS) in patients with ccRCC.

Various cell types express kindlin‐2, including fibroblasts, muscle cells, endothelial cells, and epithelial cells 9, 21. Expression of kindlin‐2 has also been studied in cancer cells. Gozgit *et al*. 13 showed that kindlin‐2 was highly expressed in aggressive human TMX2‐28 breast cancer cells; Kato *et al*. 12 reported that kindlin‐2 expression was increased in leiomyomas compared with the normal myometrium; Shen *et al*. 17 found that kindlin‐2 was upregulated at both RNA and protein levels in gastric cancer tissues. Previous studies have suggested that high levels of kindlin‐2 might contribute to tumor invasion 15. Yu *et al*. 20 reported that kindlin‐2 may promote tumor cell invasion by selectively strengthening the occupancy of β‐catenin on the Wnt target gene Axin2, thereby enhancing Axin2 gene expression. Suppression of kindlin‐2 expression significantly reduced the invasion of TMX2‐28 cells in breast cancer 13. However, a study of SK‐LMS‐1 leiomyosarcoma and HT‐1080 fibrosarcoma demonstrated that knockdown of kindlin‐2 markedly increased the invasiveness of these cells 14. Our work suggests that high kindlin‐2 levels are likely to contribute to the invasiveness of ccRCC. We searched the TCGA database and found that kindlin‐2 was upregulated in ccRCC at the RNA level, although the potential mechanism by which kindlin‐2 regulates the metastasis of ccRCC is still unclear. Kindlin‐2 may affect tumor cell proliferation and invasion by activating the Wnt signaling pathway 16, and its over expression may also promote epithelial–mesenchymal transition of tumor cells.

This study has a couple of limitations. First, the relatively small sample size may limit the statistical relevance of the conclusions. Second, this study did reveal why kindlin‐2 could promote invasion of ccRCC. Further investigation with larger sample size is required to explore the underlying mechanism by which kindlin‐2 expression enhances the ability of ccRCC cells to invade and metastasize. In conclusion, this study provides further evidence that high kindlin‐2 expression is likely to increase the invasiveness of ccRCC and is associated with poor prognosis of this malignancy. These findings will help to gain new mechanistic insights into the regulation of ccRCC metastasis.

## Author contributions

MY, LZ, YW, LG, WY and JL carried out the laboratory work, technical support, and data analysis. YC and XJ conceived the study, were partially involved in the supervision of the laboratory work, and drafted the manuscript.
